# Third trimester phthalate exposure is associated with DNA methylation of growth-related genes in human placenta

**DOI:** 10.1038/srep33449

**Published:** 2016-09-22

**Authors:** Yan Zhao, Jiao Chen, Xiu Wang, Qi Song, Hui-Hui Xu, Yun-Hui Zhang

**Affiliations:** 1 Key Laboratory of Public Health Safety, Ministry of Education, School of Public Health, Fudan University, Shanghai, China; 2 Shanghai Municipal Center for Disease Control & Prevention, Shanghai, China

## Abstract

Strong evidence implicates maternal phthalate exposure during pregnancy in contributing to adverse birth outcomes. Recent research suggests these effects might be mediated through the improper regulation of DNA methylation in offspring tissue. In this study, we examined associations between prenatal phthalate exposure and DNA methylation in human placenta. We recruited 181 mother-newborn pairs (80 fetal growth restriction newborns, 101 normal newborns) in Wenzhou, China and measured third trimester urinary phthalate metabolite concentrations and placental DNA methylation levels of *IGF2* and *AHRR*. We found urinary concentrations of mono (2-ethyl-5- hydroxyhexyl) phthalate (MEHHP), and mono (2-ethyl-5-oxohexyl) phthalate (MEOHP) were significantly inversely associated with placental *IGF2* DNA methylation. The associations were much more evident in fetal growth restriction (FGR) newborns than those in normal newborns. These findings suggest that changes in placental DNA methylation might be part of the underlying biological pathway between prenatal phthalate exposure and adverse fetal growth.

Maternal exposure to phthalates during pregnancy has been associated with higher risk of adverse pregnancy outcomes, including preterm delivery^1^,^2^, low birth weight (LBW)^3^, and intrauterine growth restriction (IUGR)^4^. Recent evidence suggests that maternal prenatal phthalate exposure affects childhood intellectual development^5^, childhood growth and blood pressure^6^. The biological mechanism of phthalate-mediated reproductive and developmental toxicity is unclear. Epigenetic modifications such as DNA methylation, may be a potential pathway linking maternal phthalate exposure to adverse effects^7^,^8^.

DNA methylation, the best characterized mechanism of epigenetic regulation, involves the covalent addition of methyl groups to cytosine to form 5-methyl-cytosine (5mC)^9^,^10^,^11^. DNA methylation plays a key role in many aspects of gene regulation, including transcriptional silencing, embryonic development, genomic imprinting, and X-chromosome inactivation^12^,^13^,^14^,^15^. Changes to DNA methylation can occur throughout life, but much of the epigenome is established during embryogenesis and early development of the fetus^16^.

Emerging data from animal studies suggest that phthalates may alter the patterns of DNA methylation. Differences in global and gene specific DNA methylation associated with phthalate exposure have been reported across various tissue types (testis, adrenal, pancreas and ovarian)^17^,^18^,^19^,^20^,^21^. However, in human study, only a few studies reported DNA methylation alterations in relation to phthalate exposure^8^,^22^,^23^.

In this present study, we measured DNA methylation of 2 growth-related genes (*IGF2* and *AHRR*) in placentas from fetal growth restriction (FGR) newborns and normal newborns. *IGF2* is a reciprocally critical imprinted gene implicated in fetal and embryonic growth^24^, and *AHRR* serves to inhibit aryl-hydrocarbon receptor transcription, which is involved in mediating xenobiotic metabolism^25^,^26^. The aim of this study was to investigate the effect of prenatal phthalate exposure on placental DNA methylation, with a focus on differences in sensitivity between FGR newborns and normal newborns. We hypothesize that changes in placental DNA methylation is part of the underlying biological pathway between prenatal phthalate exposure and adverse fetal growth.

## Methods

### Study Population

Study subjects were from a nested case-control study, which was conducted in Wenzhou to investigate the association between prenatal environmental endocrine disruptors (EDCs) exposure and FGR. The study design and protocols are described elsewhere^27^. In briefly, 220 mother-newborn pairs, including 110 FGR newborns and 110 normal newborns and their mothers, were enrolled in the Second Affiliated Hospital of Wenzhou Medical College from December 2011 to November 2013. The study was approved by Fudan University’s Institutional Review Board. All participants provided written informed consents before study. All methods were carried out in accordance with the approved guidelines.

Questionnaire information concerning maternal weight, and height before pregnancy, maternal age, maternal smoking, drinking, dietary habits, etc., was collected by interview. Birth outcomes (birth weight, birth length and head circumference,) were extracted from hospital records. Gestational age was assigned using reported date of last menstrual period.

Maternal urine samples were collected during the third trimester. For a better comparability of urine phthalate metabolites among individuals, we collected first morning urine. Urine samples were stored at −20 °C until analysis. Placenta samples were collected immediately after delivery. For each subject, eight biopsies of apparently normal tissue were collected (two from each of the four quadrants). All samples were taken from the maternal side of the placenta, 2 cm from the umbilical cord insertion site, free of maternal decidua. Placenta samples were stored at −80 °C until analysis.

### Urinary Phthalate metabolites Measurement

Urinary concentrations of 5 phthalate ester metabolites were measured. Two lower-molecular-weight phthalate metabolites are mono-n-butyl phthalate (MBP) and monomethyl phthalate (MMP). MBP and MMP are the primary metabolite of dibutyl phthalate (DBP) and dimethyl phthalate (DMP), respectively. Three higher-molecular-weight phthalate metabolites are mono-2-ethylhexyl phthalate (MEHP), mono (2-ethyl-5-oxohexyl) phthalate (MEOHP) and mono (2-ethyl-5-hydroxyhexyl) phthalate (MEHHP). MEHP is the primary metabolite of DEHP, and MEHHP, MEOHP are secondary metabolites of DEHP^28^.

Urinary phthalate metabolites concentrations were analyzed using analytical methods described in detail elsewhere^4^,^29^. Briefly, the phthalate metabolites were first enzymatically deconjugated and then extracted from the urine using solid-phase extraction, separated by an Agilent 1100 Series high-performance liquid chromatography (HPLC) system, and detected by an API 2000 electrospray triple quadrupole mass spectrometer (ESI-MS/MS; Applied Biosystems, Foster City, CA). In each analytical run, internal standards, method blanks and quality control samples were used to increase the accuracy and precision of the measurements. The limits of detection were 0.25 ng/mL for MMP and 0.50 ng/mL for MBP, MEHP, MEOHP and MEHHP.

Because of the glucuronidation of phthalate metabolites in the liver and its elimination by active tubular secretion, creatinine correction may not be appropriate for urinary phthalate metabolite concentration^30^,^31^. Thus, urinary phthalate levels were normalized for dilution by specific gravity, as recommended by Hauser *et al.*^31^. The correction formula was Pc = P × [(1.024 − 1)/(SG-1)], where Pc is the specific gravity-corrected phthalate metabolite concentration (ng/mL), P is the observed phthalate concentration (ng/mL), and SG is the specific gravity of the urine sample. Specific gravity was measured using a handheld refractometer (Atago PAL 10-S, Tokyo, Japan), which was calibrated with deionized water before each measurement^32^.

### DNA extraction and Bisulfite modification

Four biopsies of each sample, two from the upper left hand quadrant and two from the lower right hand quadrant, were pooled and then used for DNA extraction. Pooled sample was rinsed twice in 1.0 ml PBS, 5 min prior to DNA extraction to remove traces of maternal blood. Genomic DNA was extracted using the QIAmp DNA Mini Kit (Qiagen, Hilden, Germany) according to the manufacturer’s protocol. Sample concentrations and purity ratios (260/230 and 260/280) were measured using a NanoDrop spectrophotometer device (Nanodrop, Wilmington, DE, USA).

A total of 500 ng DNA was modified by treatment with sodium bisulfite using the Zymo EZ DNA Methylation kit (Zymo Research, Orange, CA, USA). Bisulfite treatment of denatured DNA converts all unmethylated cytosines to uracils but leaves methylated cytosines unchanged, allowing quantitative definition of cytosine methylation status. Bisulfite-converted DNA was eluted in 30 μl M-Elution buffer and stored at −20 °C until analysis.

### PCR and Pyrosequencing for placental DNA methylation

We performed DNA methylation analysis on bisulfite-treated DNA using a quantitative assay based on PCR-pyrosequencing. PCR and pyrosequencing primers have been previously published^27^,^33^,^34^ and have been shown in Table S1. Bisulfite-convert DNA was amplified using GoTaqR Hot Start Polymerase (Promega, Madison, USA). The PCR product underwent pyrosequencing using PyroMak Q96 MD Pyrosequencing System (Qiagen, Germany). Methylation level of each CG dinucleotide was expressed as methylated cytosines over the sum of methylated and unmethylated cytosines. Each sample was tested in triplicate (starting with the bisulfite conversion) and the average was used for statistical analysis.

Some quality controls were included in every pyrosequencing run. A human unmethylated (0%) standard and fully methylated (100%) standard were used as sample controls. To verify bisulfite conversion efficiency, a C outside a CG site was used in every assay as built-in control. To ensure that pyrosequencing sequenced the correct pattern, two wells were filled with oligonucleotide with a known sequence.

### Statistical analysis

Specific gravity-corrected phthalate metabolite concentration was used in data analysis. For phthalate metabolite concentrations below the LOD, we replaced them with one-half of the LOD. As urinary phthalate metabolite concentrations were not normally distributed, medians were presented to characterize urinary phthalate metabolite concentrations in the descriptive analysis. In addition to 5 individual phthalate metabolites, concentrations of DEHP metabolites MEHP, MEHHP and MEOHP were summed as total DEHP (SumDEHP). Differences in phthalate metabolite concentrations between FGR and control group were evaluated by Mann-Whitney *U*-test. Methylation levels of 2 CpG sites in *IGF2* and 3 CpG sites in *AHRR* were evaluated and expressed as 5 mc%. Percent methylation was used as a continuous variable. Potential differences in *IGF2* and *AHRR* methylation between FGR and control groups were tested through Mann-Whitney *U*-test.

Multiple linear regression models were used to model the associations between placental DNA methylation and phthalate exposure. Methylation level was modeled as a function of log-transformed phthalates urinary concentration, adjusting for relevant covariates. Covariates, including infant gestational, infant gender, maternal age, pre-pregnancy BMI, infection, prenatal vitamin use, maternal environmental tobacco smoke (ETB) in pregnancy, maternal alcohol use in pregnancy, maternal education and monthly income were considered as potential confounders and were retained in the model if they were significant at P < 0.20. In addition to the regression models including all the subjects, stratified modeling by birth outcome status (FGR newborns or control newborns) was also conducted. The regression coefficient (β) represented the change of DNA methylation per one unit of log-transformed urine phthalate metabolite concentration. All statistical analyses were conducted using the SPSS 16.0 statistical package (SPSS Inc., Chicago, IL, USA). P < 0.05 was considered significant, and all statistical tests were two-sided.

## Results

### Characteristics of the subjects

During the study period, 181 subjects provided both urinary and placenta samples, thus those 181 subjects, including 80 FGR cases and 101 normal controls, were enrolled in this present study. The maternal and pregnancy characteristics of FGR cases and controls were previously reported^27^. Cases and controls differed with regard to fetal birth weight, fetal birth length and GA. Cases and controls did not differ by maternal age, birth type, infant sex, and maternal pre-pregnant BMI.

### Urinary concentrations of phthalate metabolites

Table 1 showed the concentrations of 5 phthalate metabolites in urine samples. Median levels with specific gravity adjustments for 5 urinary phthalate metabolites ranged from 4.2 to 25.7 ng/ml. Levels of urinary MBP were the highest of the 5 metabolites measured. Analysis of variance showed that urinary concentrations of MEHHP, MEOHP and SumDEHP were significant higher in FGR cases than those in normal controls.

### Placental DNA methylation levels

**Figure 1:**
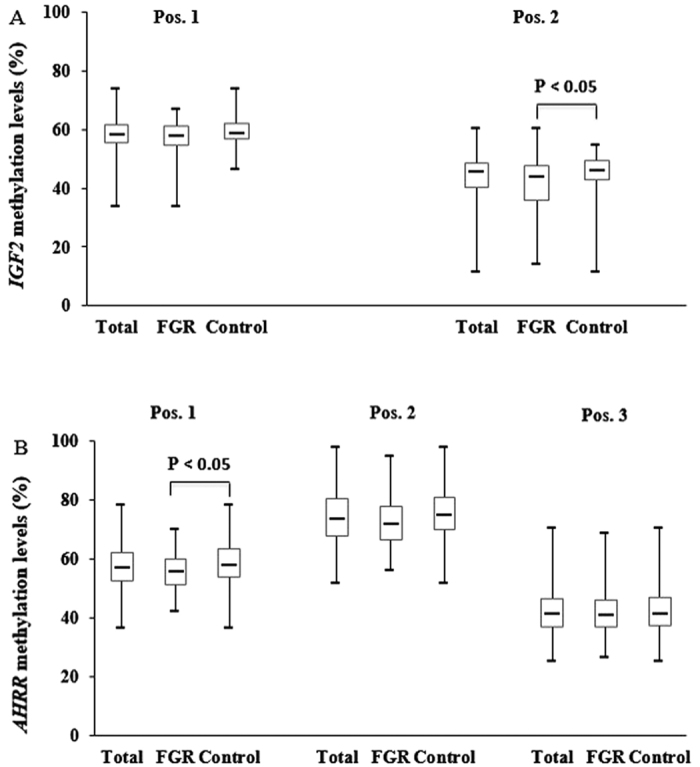
Placental DNA methylation levels of *IGF2* and *AHRR*. (**A**) *IGF2* methylation levels. (**B**) *AHRR* methylation levels. Solid bar indicates median; upper and lower limits of box, 75^th^ and 25^th^ percentiles; upper and lower bars, maximum and minimum values, respectively.

Figure 1 showed DNA methylation levels of *IGF2* and *AHRR*. The median methylation levels of *IGF2* were 58.55% for position 1 and 45.77% for position 2. In comparison, the median methylation levels of *AHRR* were 57.20% for position 1, 73.78% for position 2, and 41.23% for position 3. Analysis of variance showed that DNA methylation at position 2 of *IGF2* and position 1 of *AHRR* was significant lower in FGR newborns than in control newborns.

### Association between placental DNA methylation and phthalate exposure

Using methylation data from our previous study^27^, we modeled the associations between placental DNA methylation and urinary phthalate metabolite concentrations, adjusting for gestational age, ETB, maternal age, delivery type, infection and infant sex. The associations between lower-molecular weight phthalate metabolite (MBP and MMP) concentrations and placental DNA methylation of *IGF2* and *AHRR* are presented in Figures S1 and S2. No significant associations were observed between placental DNA methylation and urinary concentrations of MBP and MMP.

**Figure 2:**
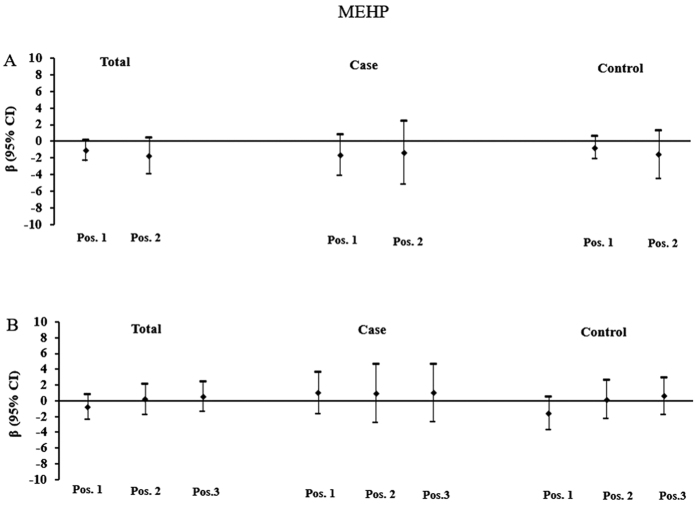
Associations of maternal urinary concentrations of MEHP with placental DNA methylation of *IGF2* and *AHRR*. (**A**) Adjusted β and 95% CI for *IGF2* DNA methylation. (**B**) Adjusted β and 95% CI for *AHRR* DNA methylation. Adjusted for gestational age, ETB, maternal age, delivery type, and infant sex, and other phthalate metabolites.

**Figure 3:**
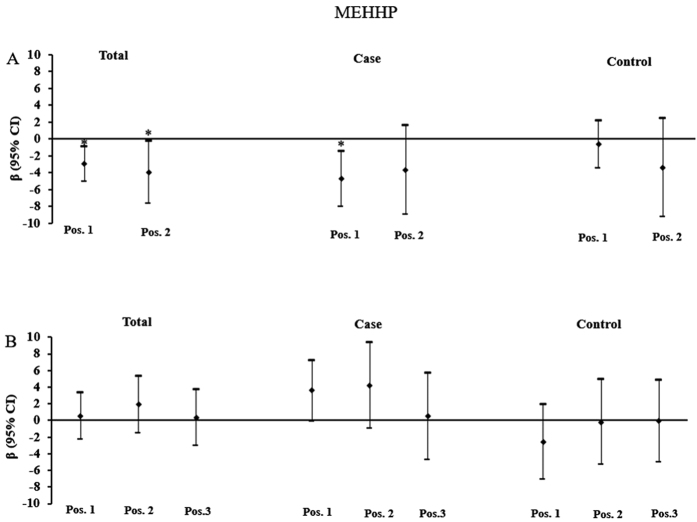
Associations of maternal urinary concentrations of MEHHP with placental DNA methylation of *IGF2* and *AHRR*. (**A**) Adjusted β and 95% CI for *IGF2* DNA methylation. (**B**) Adjusted β and 95% CI for *AHRR* DNA methylation. Adjusted for gestational age, ETB, maternal age, delivery type, and infant sex, and other phthalate metabolites. *P < 0.05.

**Figure 4:**
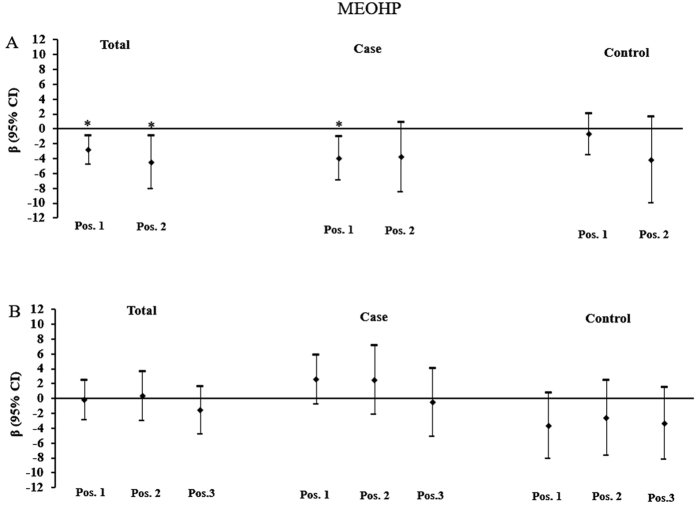
Associations of maternal urinary concentrations of MEOHP with placental DNA methylation of *IGF2* and *AHRR*. (**A**) Adjusted β and 95% CI for *IGF2* DNA methylation. (**B**) Adjusted β and 95% CI for *AHRR* DNA methylation. Adjusted for gestational age, ETB, maternal age, delivery type, and infant sex, and other phthalate metabolites. *P < 0.05.

Figures 2, 3 and 4 depicted the relationship between higher-molecular weight phthalate metabolite (MEHP, MEOHP and MEHHP) concentrations and placental DNA methylation of *IGF2* and *AHRR*. In all subjects, urinary concentrations of MEOHP and MEHHP were significantly inversely associated with DNA methylation at position 1 and position 2 of *IGF2.* A log-unit (10-fold) increase in urinary concentration of MEHHP was associated with 2.95% (position 1: β = −2.953, P = 0.005) and 3.92% (position 2: β = −3.923, P = 0.039) decrease in *IGF2* methylation. In comparison, a log-unit (10-fold) increase in MEOHP concentration was associated with 2.88% (position 1: β = −2.879, P = 0.004) and 4.52% (position 2: β = −4.518, P = 0.013) decrease in *IGF2* methylation. When subjects were stratified by birth outcome status, MEHHP and MEOHP were still found to be inversely associated DNA methylation at position 1 of *IGF2* in FGR newborns. While in normal newborns, all significant associations disappeared.

**Figure 5:**
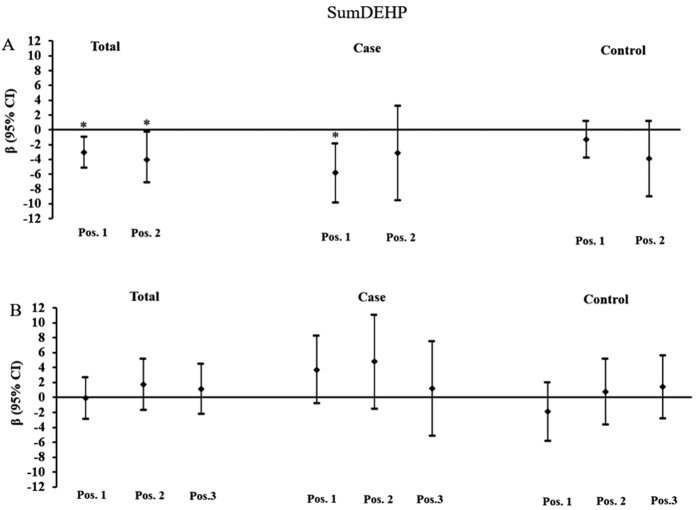
Associations of maternal urinary concentrations of SumDEHP with placental DNA methylation of *IGF2* and *AHRR*. (**A**) Adjusted β and 95% CI for *IGF2* DNA methylation. (**B**) Adjusted β and 95% CI for *AHRR* DNA methylation. Adjusted for gestational age, ETB, maternal age, delivery type, and infant sex, and other phthalate metabolites. *P < 0.05.

In addition to the individual phthalate metabolite, the total concentrations of DEHP metabolites (SumDEHP) were also correlated with placental DNA methylation (Fig. 5). Similar to MEHHP and MEOHP, elevated urinary concentrations of SumDEHP were significant associated with decreased DNA methylation at position 1 and position 2 of *IGF2* in all subjects. Among FGR newborns, there was a consistent positive association between SumDEHP concentration and DNA methylation at position 1 of *IGF2*. While among normal newborns, no significant association was observed.

## Discussion

Proper epigenetic regulation of both imprinted and non-imprinted genes is important in placental and fetal development^35^. We and others have previously reported associations between *IGF2* and *AHRR* methylation and measures of fetal growth^27^,^33^,^34^. Since prenatal phthalate exposure is negatively associated with fetal growth^3^,^4^, it is logical to explore how third trimester phthalate exposure correlates with *IGF2* and *AHRR* methylation in placenta samples.

In this study, the median concentrations of third trimester urinary phthalate metabolites ranged from 4.2 to 25.7 ng/ml. Compared with the results reported in urine samples collected from pregnant women in Germany^36^, Israel^37^, Japan^38^, Norway^39^, Sweden^40^, and the United States^41^, concentrations of MBP and MMP were comparable. However, the concentrations of DEHP metabolites (MEHP, MEHHP and MEOHP) in our study were somewhat lower.

We observed decreased *IGF2* DNA methylation in association with elevated urinary phthalate metabolite concentrations. *IGF2* is the most intensively studied imprinted gene and has been shown to play a fundamental role in regulating placental and fetal growth^24^. Consistent with our study, a recent study analyzing the same CpG sites of *IGF2*, showed placental DNA methylation levels of *IGF2* were significantly inversely associated with first trimester urinary concentrations of phthalate metabolites^23^.

*AHRR*, the aryl-hydrocarbon receptor repressor gene, is a known cancer susceptibility gene. *AHRR* methylation is particularly interesting to study during pregnancy because the aryl-hydrocarbon receptor (AHR) is involved in metabolizing xenobiotics that might affect fetal development^33^,^42^. Several studies have reported the effect of maternal smoking during pregnancy on offspring DNA methylation of *AHRR*^43^,^44^,^45^. However, in our study, we did not observe the effect of prenatal phthalate exposure on *AHRR* methylation in human placenta.

The mechanism by which phthalates interfere with DNA methylation remains unclear. Phthalates are known to increase the production of reactive oxygen species^46^,^47^. Oxidative DNA damage can inhibit binding of methyl-CpG binding proteins and alter DNA methyltransferase function^48^. We speculate that phthalate-induced oxidative stress reduced the fidelity of the epigenetic machinery, resulting in hypomethylation of cytosine residues.

This study, to our knowledge, is the first study to report alterations in placental *IGF2* methylation in relation to third trimester phthalate exposure. Our finding adds to the evidence that prenatal phthalate exposure influences DNA methylation in the placenta. The methylation marker presented here might be intended as molecular biomarker of phthalate exposure and can be used to assess phthalate-associated adverse birth outcomes.

A considerable strength of this study is that we used the population-based ascertainment of placental sample, which is a target tissue directly responsible for fetal growth. We found the associations of placental *IGF2* methylation with phthalate exposure were much more evident in FGR newborns than that in normal newborns, which indicate that placental *IGF2* methylation might mediate the association between prenatal phthalate exposure and the development of FGR. The limitations of this study include the relatively small sample size, which may restrict our statistical power. Additionally, we chose to focus on *IGF2* and *AHRR*, we could not exclude that DNA methylation of additional growth-related genes are involved linking prenatal phthalate exposure and fetal growth.

In summary, we found placental DNA methylation is differentially associated with prenatal phthalate exposure based on birth outcome status. Our work suggests that changes in placental DNA methylation might be part of the underlying biological pathway between prenatal phthalate exposure and adverse fetal growth.

## Additional Information

**How to cite this article**: Zhao, Y. *et al.* Third trimester phthalate exposure is associated with DNA methylation of growth-related genes in human placenta. *Sci. Rep.* **6**, 33449; doi: 10.1038/srep33449 (2016).

## Supplementary Material

Supplementary Information

## Figures and Tables

**Table 1:** Distribution of urinary phthalates metabolites (ng/ml).

Phthalate	Total (N = 181)^a^	FGR(N = 80)^a^	Control (N = 101)^a^	p-value^b^
MBP	25.7(11.4, 45.8)	29.5(14.7, 47.2)	21.3(9.7, 44.7)	0.116
MMP	8.1(4.5, 15.3)	8.3(5.4, 19.6)	7.9(3.8, 13.9)	0.068
MEHP	3.8(0.9, 12.9)	5.6(1.5, 12.7)	3.0(0.7, 14.0)	0.224
MEHHP	10.8(4.0, 19.2)	14.9(5.9, 26.9)	9.4(3.1, 16.8)	0.003**
MEOHP	4.2(1.6, 9.6)	5.7(1.9, 11.6)	3.7(1.0, 6.8)	0.008**
SumDEHP ^c^	25.5(12.9, 44.3)	29.0(14.7, 49.5)	20.4(9.0, 39.3)	0.025*

^a^Values are median (range). ^b^p-value calculated using the Mann-Whitney-U test. ^c^Sum of MEHP, MEHHP and MEOHP. **p < 0.01, *p < 0.05.
