# Supplementary material for: Third trimester phthalate exposure is associated with DNA methylation of growth-related genes in human placenta
Source: Sci Rep. 2016 Sep 22;6:33449. doi: 10.1038/srep33449 (PMC5031987; doi:10.1038/srep33449)
Supplement: Supplementary Information [file srep33449-s1.pdf]

Third trimester phthalate exposure is associated with DNA methylation of growth-related genes in human placenta

Yan Zhao,[1] Jiao Chen,[1] Xiu Wang,[1] Qi Song,[1] Huihui Xu,[2*] Yunhui Zhang [1*]

[1]Key Laboratory of Public Health Safety, Ministry of Education, School of Public Health, Fudan University, Shanghai, China

[2] Shanghai Municipal Center for Disease Control & Prevention, Shanghai, China

**Gene**	**PCR primer**	**CpGs position**
IGF2	Primer Forward (Bio): ATGAATGAGTATTTTTAGGGAAATTGTT Primer Reverse: TCCATATCCCCCCTAAATTTAACTTCT Sequencing primer: CCTCCCTAACACAAAA	Pos1=2148430 Pos2=2148479
AHRR	Primer Forward: GGTAGTTATTTAGTTAAGTTTTTTTT Primer Reverse (Bio): TTCACTCTAAATACTAAAACATTTC Sequencing primer: TAAGTTTTTTTTTGA	Pos1=420093 Pos2=420109 Pos3=420119

Table S1. PCR and Pyrosequencing primers.

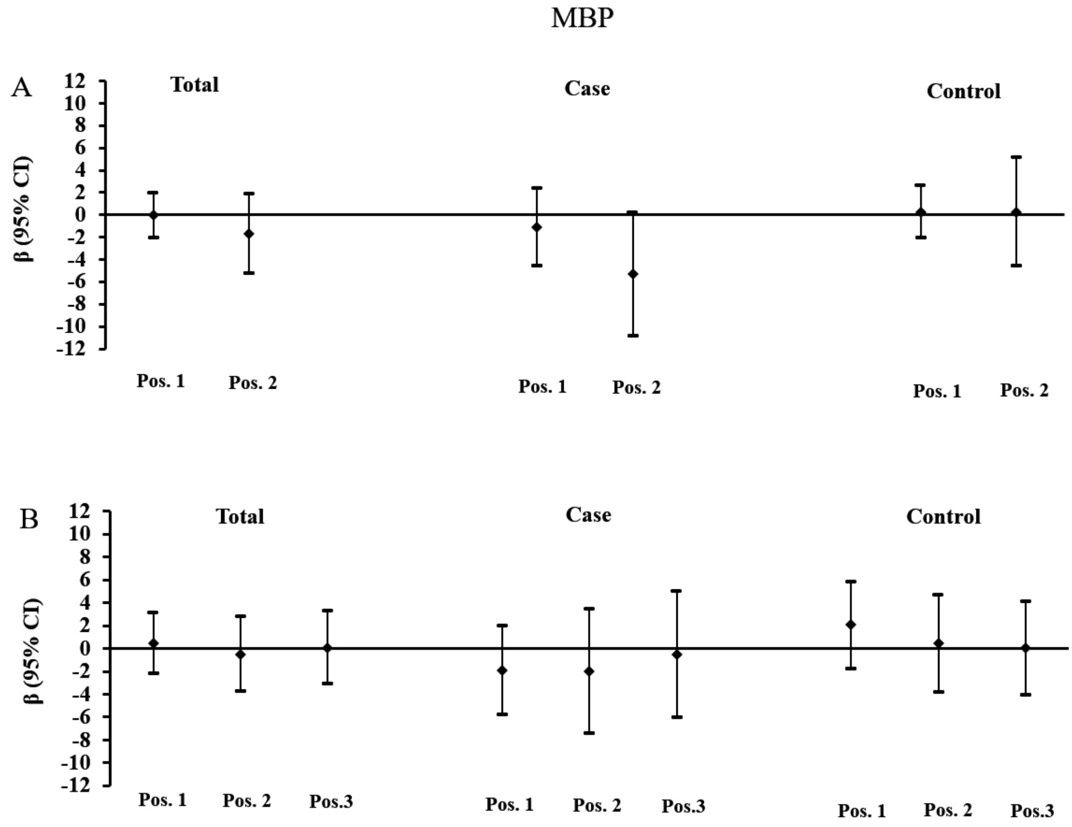

Figure S1. Associations of maternal urinary concentrations of MBP with placental DNA methylation of *IGF2* and *AHRR*. (**A**) Adjusted β and 95% CI for *IGF2* DNA methylation. (**B**) Adjusted β and 95% CI for *AHRR* DNA methylation. Adjusted for gestational age, ETB, maternal age, delivery type, and infant sex, infection, and other phthalate metabolites.

MMP

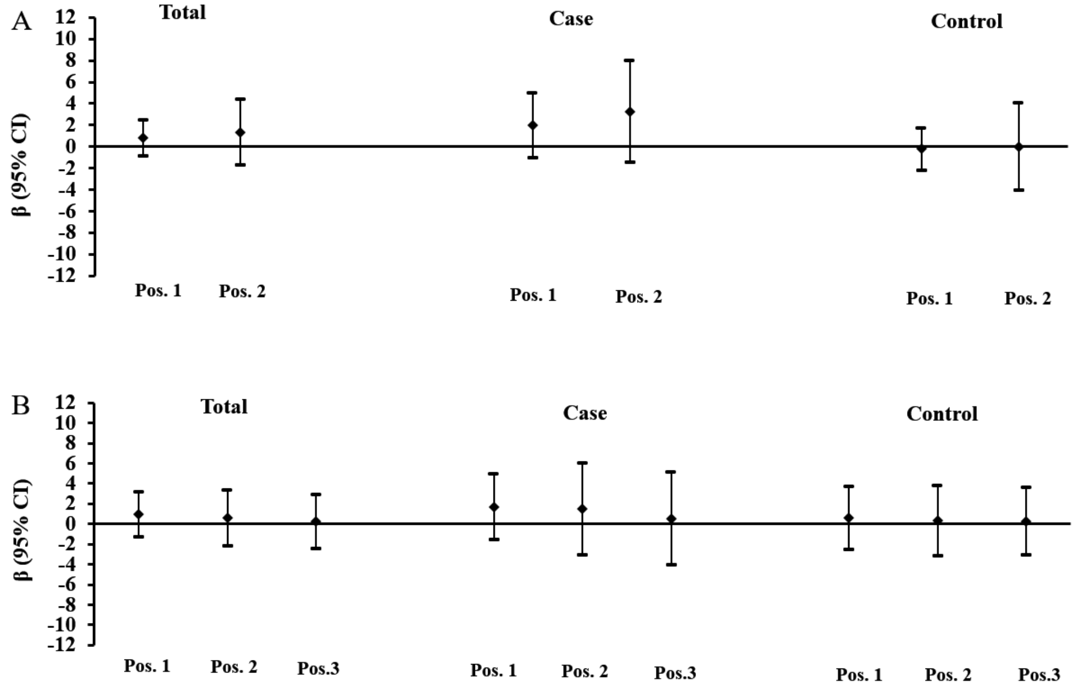

Figure S2. Associations of maternal urinary concentrations of MMP with placental DNA methylation of *IGF2* and *AHRR*. (**A**) Adjusted β and 95% CI for *IGF2* DNA methylation. (**B**) Adjusted β and 95% CI for *AHRR* DNA methylation. Adjusted for gestational age, ETB, maternal age, delivery type, and infant sex, infection and other phthalate metabolites.
